# Supplementary material for: Concurrent credit portfolio losses
Source: PLoS One. 2018 Feb 9;13(2):e0190263. doi: 10.1371/journal.pone.0190263 (PMC5806874; doi:10.1371/journal.pone.0190263)
Supplement: S1 Appendix — (PDF) [file pone.0190263.s001.pdf]

S1 Appendix. Download of financial data from Yahoo! finance. In the sequel, we give the lists of the ticker symbols and the corresponding companies for the S&P 500 and Nikkei 225 data which we used in our analysis as they appear on the webpage Yahoo! finance, see Ref. [29] in the main text. This webpage provides a webform, on which the ticker symbol has to be typed into a text box, and the stock exchange has to be chosen from a select box, in our case New York Stock Exchange (NYSE) for the S&P 500 data and Tokio Stock Exchange (TSE) for the Nikkei 225 data. A click on the button “historical data” leads to the time series to be analyzed, the desired time period, in our case 01/1993–04/2014, is specified via the “time period” button. Using the “download data” button finally starts the download of the data.

Ticker symbols and corresponding companies in S&P 500

MMM, 3M Co
ABT, Abbott Laboratories
ADBE, Adobe Systems Inc
AFL, Aflac Inc
GAS, AGL Resources Inc
APD, Air Products & Chemicals Inc
ARG, Airgas Inc
AA, Alcoa Inc
AGN, Allergan Inc/United States
ALTR, Altera Corp
MO, Altria Group Inc
AEP, American Electric Power Co Inc
AXP, American Express Co
AIG, American International Group Inc
AMGN, Amgen Inc
APC, Anadarko Petroleum Corp
AON, Aon PLC
APA, Apache Corp
AAPL, Apple Inc
AMAT, Applied Materials Inc
ADM, Archer-Daniels-Midland Co
T, AT& T Inc
ADSK, Autodesk Inc
AZO, AutoZone Inc
AVY, Avery Dennison Corp
AVP, Avon Products Inc
BHI, Baker Hughes Inc
BLL, Ball Corp
BAC, Bank of America Corp
BK, Bank of New York Mellon Corp/The
BAX, Baxter International Inc
BBT, BB& T Corp
BDX, Becton Dickinson and Co
BBBY, Bed Bath & Beyond Inc
BMS, Bemis Co Inc
BBY, Best Buy Co Inc
BIIB, Biogen Idec Inc
BA, Boeing Co/The
BSX, Boston Scientific Corp

BMY, Bristol-Myers Squibb Co
COG, Cabot Oil & Gas Corp
CPB, Campbell Soup Co
CCL, Carnival Corp
CAT, Caterpillar Inc
CELG, Celgene Corp
CNP, CenterPoint Energy Inc
CTL, CenturyLink Inc
CERN, Cerner Corp
CVX, Chevron Corp
CB, Chubb Corp/The
CI, Cigna Corp
CINF, Cincinnati Financial Corp
CTAS, Cintas Corp
CSCO, Cisco Systems Inc
C, Citigroup Inc
CLX, Clorox Co/The
CMS, CMS Energy Corp
KO, Coca-Cola Co/The
CCE, Coca-Cola Enterprises Inc
CL, Colgate-Palmolive Co
CMCSA, Comcast Corp
CMA, Comerica Inc
CSC, Computer Sciences Corp
CAG, ConAgra Foods Inc
COP, ConocoPhillips
ED, Consolidated Edison Inc
GLW, Corning Inc
BCR, CR Bard Inc
CSX, CSX Corp
CMI, Cummins Inc
CVS, CVS Caremark Corp
DHR, Danaher Corp
DE, Deere & Co
XRAY, DENTSPLY International Inc
D, Dominion Resources Inc/VA
DOV, Dover Corp
DOW, Dow Chemical Co/The
DTE, DTE Energy Co
DUK, Duke Energy Corp
ETN, Eaton Corp PLC
ECL, Ecolab Inc
EIX, Edison International
DD, EI du Pont de Nemours & Co
EA, Electronic Arts Inc
LLY, Eli Lilly & Co
EMC, EMC Corp/MA
EMR, Emerson Electric Co
ETR, Entergy Corp
EOG, EOG Resources Inc
EQT, EQT Corp
EFX, Equifax Inc

EXC, Exelon Corp
EXPD, Expeditors International of Washington I
ESRX, Express Scripts Holding Co
XOM, Exxon Mobil Corp
FDO, Family Dollar Stores Inc
FAST, Fastenal Co
FDX, FedEx Corp
FITB, Fifth Third Bancorp
FISV, Fiserv Inc
FMC, FMC Corp
F, Ford Motor Co
BEN, Franklin Resources Inc
GCI, Gannett Co Inc
GPS, Gap Inc/The
GD, General Dynamics Corp
GE, General Electric Co
GIS, General Mills Inc
GPC, Genuine Parts Co
GILD, Gilead Sciences Inc
GHC, Graham Holdings Co
HRB, H& R Block Inc
HAL, Halliburton Co
HOG, Harley-Davidson Inc
HAR, Harman International Industries Inc
HRS, Harris Corp
HCP, HCP Inc
HCN, Health Care REIT Inc
HP, Helmerich & Payne Inc
HSY, Hershey Co/The
HES, Hess Corp
HPQ, Hewlett-Packard Co
HD, Home Depot Inc/The
HON, Honeywell International Inc
HRL, Hormel Foods Corp
HUM, Humana Inc
HBAN, Huntington Bancshares Inc/OH
ITW, Illinois Tool Works Inc
IR, Ingersoll-Rand PLC
TEG, Integrys Energy Group Inc
INTC, Intel Corp
IBM, International Business Machines Corp
IFF, International Flavors & Fragrances Inc
IGT, International Game Technology
IP, International Paper Co
IPG, Interpublic Group of Cos Inc/The
JEC, Jacobs Engineering Group Inc
SJM, JM Smucker Co/The
JNJ, Johnson & Johnson
JCI, Johnson Controls Inc
JPM, JPMorgan Chase & Co
KSU, Kansas City Southern
K, Kellogg Co

KEY, KeyCorp
KMB, Kimberly-Clark Corp
KIM, Kimco Realty Corp
KLAC, KLA-Tencor Corp
KSS, Kohl's Corp
KR, Kroger Co/The
LB, L Brands Inc
LRCX, Lam Research Corp
LM, Legg Mason Inc
LEG, Leggett & Platt Inc
LEN, Lennar Corp
LUK, Leucadia National Corp
LNC, Lincoln National Corp
LLTC, Linear Technology Corp
L, Loews Corp
LOW, Lowe's Cos Inc
M, Macy's Inc
MRO, Marathon Oil Corp
MMC, Marsh & McLennan Cos Inc
MAS, Masco Corp
MCD, McDonald's Corp
MHFI, McGraw Hill Financial Inc
MDT, Medtronic Inc
MRK, Merck & Co Inc
MSFT, Microsoft Corp
MSI, Motorola Solutions Inc
MUR, Murphy Oil Corp
NWL, Newell Rubbermaid Inc
NEM, Newmont Mining Corp
NEE, NextEra Energy Inc
NKE, NIKE Inc
NI, NiSource Inc
NBL, Noble Energy Inc
NSC, Norfolk Southern Corp
NU, Northeast Utilities
NTRS, Northern Trust Corp
NOC, Northrop Grumman Corp
NUE, Nucor Corp
OXY, Occidental Petroleum Corp
OMC, Omnicom Group Inc
OKE, ONEOK Inc
OI, Owens-Illinois Inc
PCAR, PACCAR Inc
PLL, Pall Corp
PH, Parker Hannifin Corp
PDCO, Patterson Cos Inc
PAYX, Paychex Inc
PBCT, People's United Financial Inc
PEP, PepsiCo Inc
PKI, PerkinElmer Inc
PFE, Pfizer Inc
PCG, PG& E Corp PNW, Pinnacle West Capital Corp

PBI, Pitney Bowes Inc
PCL, Plum Creek Timber Co Inc
PNC, PNC Financial Services Group Inc/The
PPG, PPG Industries Inc
PPL, PPL Corp
PX, Praxair Inc
PCP, Precision Castparts Corp
PG, Procter & Gamble Co/The
PGR, Progressive Corp/The
PEG, Public Service Enterprise Group Inc
PHM, PulteGroup Inc
PVH, PVH Corp
QCOM, QUALCOMM Inc
RTN, Raytheon Co
REGN, Regeneron Pharmaceuticals Inc
RHI, Robert Half International Inc
ROST, Ross Stores Inc
RDC, Rowan Cos Plc
R, Ryder System Inc
SWY, Safeway Inc
SCG, SCANA Corp
SLB, Schlumberger Ltd
SHW, Sherwin-Williams Co/The
SIAL, Sigma-Aldrich Corp
SNA, Snap-on Inc
SO, Southern Co/The
LUV, Southwest Airlines Co
SWN, Southwestern Energy Co
SWK, Stanley Black & Decker Inc
SPLS, Staples Inc
SBUX, Starbucks Corp
HOT, Starwood Hotels & Resorts Worldwide Inc
STI, SunTrust Banks Inc
SYMC, Symantec Corp
SYY, Sysco Corp
TROW, T Rowe Price Group Inc
TGT, Target Corp
TE, TECO Energy Inc
THC, Tenet Healthcare Corp
TSO, Tesoro Corp
TXT, Textron Inc
TMO, Thermo Fisher Scientific Inc
TIF, Tiffany & Co
TJX, TJX Cos Inc/The
TMK, Torchmark Corp
TSS, Total System Services Inc
TRV, Travelers Cos Inc/The
TYC, Tyco International Ltd
UNP, Union Pacific Corp
X, United States Steel Corp
UTX, United Technologies Corp
UNH, UnitedHealth Group Inc

UNM, Unum Group
USB, US Bancorp/MN
VAR, Varian Medical Systems Inc
VTR, Ventas Inc
VZ, Verizon Communications Inc
VRTX, Vertex Pharmaceuticals Inc
VFC, VF Corp
VNO, Vornado Realty Trust
VMC, Vulcan Materials Co
WMT, Wal-Mart Stores Inc
WAG, Walgreen Co
DIS, Walt Disney Co/The
WFC, Wells Fargo & Co
WY, Weyerhaeuser Co
WHR, Whirlpool Corp
WFM, Whole Foods Market Inc
WMB, Williams Cos Inc/The
WEC, Wisconsin Energy Corp
GWW, WW Grainger Inc
XEL, Xcel Energy Inc
XRX, Xerox Corp
XLNX, Xilinx Inc
XL, XL Group PLC
ZION, Zions Bancorporation

Ticker symbols and corresponding companies in Nikkei 225

6857, Advantest Corp
8267, Aeon Co Ltd
2802, Ajinomoto Co Inc
6770, Alps Electric Co Ltd
6113, Amada Co Ltd
9202, ANA Holdings Inc
5201, Asahi Glass Co Ltd
2502, Asahi Group Holdings Ltd
3407, Asahi Kasei Corp
4503, Astellas Pharma Inc
8332, Bank of Yokohama Ltd/The
5108, Bridgestone Corp
7751, Canon Inc
6952, Casio Computer Co Ltd
8331, Chiba Bank Ltd/The
6366, Chiyoda Corp
9502, Chubu Electric Power Co Inc
4519, Chugai Pharmaceutical Co Ltd
7762, Citizen Holdings Co Ltd
8253, Credit Saison Co Ltd
7912, Dai Nippon Printing Co Ltd
6367, Daikin Industries Ltd
7735, Dainippon Screen Manufacturing Co Ltd
4506, Dainippon Sumitomo Pharma Co Ltd
1925, Daiwa House Industry Co Ltd

8601, Daiwa Securities Group Inc
4061, Denki Kagaku Kogyo KK
6902, Denso Corp
5714, Dowa Holdings Co Ltd
6361, Ebara Corp
4523, Eisai Co Ltd
6954, FANUC Corp
6504, Fuji Electric Co Ltd
7270, Fuji Heavy Industries Ltd
4901, FUJIFILM Holdings Corp
5803, Fujikura Ltd
6702, Fujitsu Ltd
5715, Furukawa Co Ltd
5801, Furukawa Electric Co Ltd
8803, Heiwa Real Estate Co Ltd
7205, Hino Motors Ltd
6305, Hitachi Construction Machinery Co Ltd
6501, Hitachi Ltd
7004, Hitachi Zosen Corp
3865, Hokuetsu Kishu Paper Co Ltd
7267, Honda Motor Co Ltd
7013, IHI Corp
7202, Isuzu Motors Ltd
8001, ITOCHU Corp
5631, Japan Steel Works Ltd/The
1963, JGC Corp
6473, JTEKT Corp
1812, Kajima Corp
9503, Kansai Electric Power Co Inc/The
4452, Kao Corp
7012, Kawasaki Heavy Industries Ltd
9107, Kawasaki Kisen Kaisha Ltd
9008, Keio Corp
9009, Keisei Electric Railway Co Ltd
2801, Kikkoman Corp
2503, Kirin Holdings Co Ltd
5406, Kobe Steel Ltd
6301, Komatsu Ltd
9766, Konami Corp
4902, Konica Minolta Inc
6326, Kubota Corp
3405, Kuraray Co Ltd
6971, Kyocera Corp
4151, Kyowa Hakko Kirin Co Ltd
8002, Marubeni Corp
8252, Marui Group Co Ltd
7261, Mazda Motor Corp
6508, Meidensha Corp
6479, Minebea Co Ltd
8058, Mitsubishi Corp
6503, Mitsubishi Electric Corp
8802, Mitsubishi Estate Co Ltd

7011, Mitsubishi Heavy Industries Ltd
9301, Mitsubishi Logistics Corp
5711, Mitsubishi Materials Corp
7211, Mitsubishi Motors Corp
8031, Mitsui & Co Ltd
4183, Mitsui Chemicals Inc
7003, Mitsui Engineering & Shipbuilding Co Ltd
8801, Mitsui Fudosan Co Ltd
5706, Mitsui Mining & Smelting Co Ltd
9104, Mitsui OSK Lines Ltd
6767, Mitsumi Electric Co Ltd
6701, NEC Corp
5333, NGK Insulators Ltd
2871, Nichirei Corp
7731, Nikon Corp
5214, Nippon Electric Glass Co Ltd
9062, Nippon Express Co Ltd
4272, Nippon Kayaku Co Ltd
2282, Nippon Meat Packers Inc
5202, Nippon Sheet Glass Co Ltd
4041, Nippon Soda Co Ltd
5401, Nippon Steel & Sumitomo Metal Corp
1332, Nippon Suisan Kaisha Ltd
9432, Nippon Telegraph & Telephone Corp
9101, Nippon Yusen KK
4021, Nissan Chemical Industries Ltd
7201, Nissan Motor Co Ltd
2002, Nisshin Seifun Group Inc
3105, Nisshinbo Holdings Inc
3110, Nitto Boseki Co Ltd
6988, Nitto Denko Corp
8604, Nomura Holdings Inc
6471, NSK Ltd
6472, NTN Corp
1802, Obayashi Corp
9007, Odakyu Electric Railway Co Ltd
3861, Oji Holdings Corp
6703, Oki Electric Industry Co Ltd
6103, OKUMA Corp
7733, Olympus Corp
9532, Osaka Gas Co Ltd
5541, Pacific Metals Co Ltd
6752, Panasonic Corp
6773, Pioneer Corp
7752, Ricoh Co Ltd
2501, Sapporo Holdings Ltd
9735, Secom Co Ltd
1928, Sekisui House Ltd
6753, Sharp Corp/Japan 1803, Shimizu Corp
4063, Shin-Etsu Chemical Co Ltd
4507, Shionogi & Co Ltd
4911, Shiseido Co Ltd

8355, Shizuoka Bank Ltd/The
4004, Showa Denko KK
5002, Showa Shell Sekiyu KK
6758, Sony Corp
4005, Sumitomo Chemical Co Ltd
8053, Sumitomo Corp
5802, Sumitomo Electric Industries Ltd
6302, Sumitomo Heavy Industries Ltd
5713, Sumitomo Metal Mining Co Ltd
5232, Sumitomo Osaka Cement Co Ltd
8830, Sumitomo Realty & Development Co Ltd
7269, Suzuki Motor Corp
5233, Taiheiyo Cement Corp
1801, Taisei Corp
6976, Taiyo Yuden Co Ltd
2531, Takara Holdings Inc
8233, Takashimaya Co Ltd
4502, Takeda Pharmaceutical Co Ltd
6762, TDK Corp
3401, Teijin Ltd
4543, Terumo Corp
9001, Tobu Railway Co Ltd
9602, Toho Co Ltd/Tokyo
5707, Toho Zinc Co Ltd
5301, Tokai Carbon Co Ltd
4043, Tokuyama Corp
9681, Tokyo Dome Corp
9501, Tokyo Electric Power Co Inc
8035, Tokyo Electron Ltd
9531, Tokyo Gas Co Ltd
8804, Tokyo Tatemono Co Ltd
9005, Tokyu Corp
7911, Toppan Printing Co Ltd
3402, Toray Industries Inc
6502, Toshiba Corp
4042, Tosoh Corp
5332, TOTO Ltd
5901, Toyo Seikan Group Holdings Ltd
3101, Toyobo Co Ltd
7203, Toyota Motor Corp
8015, Toyota Tsusho Corp
4208, Ube Industries Ltd/Japan
3103, Unitika Ltd
8270, UNY Group Holdings Co Ltd
7951, Yamaha Corp
9064, Yamato Holdings Co Ltd
6506, Yaskawa Electric Corp
6841, Yokogawa Electric Corp
5101, Yokohama Rubber Co Ltd/The
